# Exercise-Induced Pulmonary Hypertension Is Associated with High Cardiovascular Risk in Patients with HIV

**DOI:** 10.3390/jcm11092447

**Published:** 2022-04-27

**Authors:** Rosalinda Madonna, Silvia Fabiani, Riccardo Morganti, Arianna Forniti, Filippo Biondi, Lorenzo Ridolfi, Riccardo Iapoce, Francesco Menichetti, Raffaele De Caterina

**Affiliations:** 1 Department of Pathology, Cardiology Division, Azienda Ospedaliera Universitaria Pisana, University of Pisa, 56124 Pisa, Italy; f.biondi9@studenti.unipi.it (F.B.); ridolfi4@studenti.unipi.it (L.R.); raffaele.decaterina@unipi.it (R.D.C.); 2 Infectious Disease Unit, Department of Clinical and Experimental Medicine, Azienda Ospedaliera Universitaria Pisana, University of Pisa, 56124 Pisa, Italy; s.fabiani@ao-pisa.toscana.it (S.F.); arianna.forniti@gmail.com (A.F.); riccardo.iapoce@ao-pisa.toscana.it (R.I.); francesco.menichetti@unipi.it (F.M.); 3 Section of Statistics, University Hospital of Pisa, 56124 Pisa, Italy; r.morganti@ao-pisa.toscana.it

**Keywords:** acquired immunodeficiency syndrome, cardiovascular risk, exercise-induced pulmonary hypertension, exercise stress echocardiography, exercise cardiopulmonary test

## Abstract

**Background and Aim:** Pulmonary hypertension (PH) at rest can be preceded by the onset of exercise-induced PH (ExPH). We investigated its association with the cardiovascular (CV) risk score in patients with human immunodeficiency virus (HIV). **Methods:** In 46 consecutive patients with HIV with low (*n* = 43) or intermediate (*n* = 3) probability of resting PH, we evaluated the CV risk score based on prognostic determinants of CV risk. Diagnosis of ExPH was made by cardiopulmonary exercise test (CPET) and exercise stress echocardiogram (ESE). **Results:** Twenty-eight % (*n* = 13) of the enrolled patients had ExPH at both CPET and ESE, with good agreement between the two methods (Cohen’s kappa = 0.678). ExPH correlated directly with a higher CV score (*p* < 0.001). Patients with a higher CV score also had lower CD4+ T-cell counts (*p* = 0.001), a faster progression to acquired immunodeficiency syndrome (*p* < 0.001), a poor immunological response to antiretroviral therapy (*p* = 0.035), higher pulmonary vascular resistance (*p* = 0.003) and a higher right atrial area (*p* = 0.006). **Conclusions:** Isolated ExPH is associated with a high CV risk score in patients with HIV. Assessment of ExPH may better stratify CV risk in patients with HIV.

## 1. Introduction

HIV infection represents a potential risk factor for pulmonary arterial hypertension (PAH) [1]. PAH is the main cause of mortality in patients with HIV [2]. Reduced pulmonary vascular reserve may be silent at rest, while it occurs with PH during exercise. The onset of exercise-induced pulmonary hypertension (ExPH), therefore, represents the first clinical sign of pulmonary vascular disease that can progress to PAH. Similar to other clinical PAH groups, a significant number of patients with HIV may have ExPH [3,4,5,6,7]. However, the prognostic value of ExPH in patients with HIV is poorly studied and Guidelines from the American College of Cardiology/American Heart Association recommend further investigation [8].

Isolated ExPH is also associated with cardiovascular (CV) events in patients with valvular or ischemic heart disease [9,10], while it is associated with clinical worsening in patients with scleroderma [11].

We have recently shown that the onset of ExPH, diagnosed by exercise stress echocardiogram (ESE), is associated with poor control of HIV infection and ExPH is associated with impaired functional capacity, as measured by the World Health Organization functional class (WHO-FC), suggesting that disease progression in the lung leads to a reduction in the vasodilatory reserve of pulmonary circulation and, consequently, PH triggered by exercise [12]. How ExPH correlates with CV risk in patients with HIV has never been previously studied. Here, we hypothesized that an increased CV risk in patients with HIV can be determined early through the work-out for ExPH by ESE and cardiopulmonary stress testing (CPET).

## 2. Methods

*Study Design and Data Collection.* We conducted a prospective, observational, cohort study of patients with HIV recruited from the Infectious Disease Clinic at Pisa University Hospital. The study complies with the Helsinki Declaration, and informed consent was obtained from all patients prior to any diagnostic test. Local investigators had full access to patient data and medical records.

All of the enrolled patients underwent evaluation of PH probability at rest by transthoracic echocardiography (TTE) [9,12], followed by ExPH assessment by transthoracic exercise stress echocardiogram (ESE) and cardiopulmonary exercise test (CPET), as detailed in the Supplementary Materials. Patients included had either a “low” PH probability at rest (*n* = 43) or an “intermediate” PH probability at rest (*n* = 3). We excluded patients with a “high” PH probability (*n* = 8). Patients were then classified as either with or without ExPH at ESE or CPET. We evaluated the CV risk score by assessing the presence/absence of the prognostic determinants of cardiovascular (CV) risk, according to the 2015 European Society of Cardiology (ESC)/European Respiratory Society (ERS) Guidelines [1] and, as detailed in the Supplementary Materials. We assigned a severity score of 1–3 to each prognostic determinant and derived an overall CV risk score [11]. We then evaluated the association of a higher CV score with the presence/absence of ExPH, and with several echocardiographic parameters [12] and immuno-virological parameters.

*Mono and 2D Transthoracic Echocardiography* was performed using a Philips iE33 echocardiograph (Philips iE33 xMATRIX echocardiography system, Andover, MA) [13]. The images were recorded in at least three cardiac cycles. Right atrial pressure (RAP) was assessed by evaluating the inferior vena cava (IVC) diameter and collapsibility during inspirium [13]. Systolic pulmonary arterial pressure (PAPs) was calculated by adding RAP to the maximum systolic pressure gradient from tricuspid regurgitation velocity (TRV). Left atrial volume index (LAVi) was calculated by Simpson’s algorithm in apical four-chamber and two-chamber view [13]. Mitral, aortic and tricuspidal regurgitations were assessed by measuring the vena contracta at apical four-chamber view.

*Stress echocardiography*. All patients underwent a semi-supine ESE performed with a 2.5-MHz duplex transducer and a Philips iE33 echocardiograph (Philips iE33 xMATRIX echocardiography system, Andover, MA, USA) on a semi-recumbent cycle ergometer (Ergoline, model 900 EL, Germany), according to the European Association of Echocardiography (EAE) Guidelines [13,14,15,16,17,18]. A detailed protocol of the echocardiographic procedures is reported in the Supplementary Materials.

*Cardiopulmonary Exercise Test.* We performed the CPET on an electronically-braked cycle ergometer, using Vmax 6200 Spectra Series software (SensorMedics, Hochberg, Germany), according to a graded, cycling workload increase protocol. The test was interrupted when one of the following symptoms or signs occurred: angina; electrocardiographic signs of myocardial ischemia or injury; an excessive blood pressure increase (systolic blood pressure ≥ 240 mmHg, diastolic blood pressure ≥ 120 mmHg); dyspnea or maximal predicted heart rate. A detailed protocol is reported in the Supplementary Materials [19,20,21].

### Statistical Analyses

Categorical data were expressed by absolute and relative frequency, and continuous data by mean and standard deviation (SD). The Chi square test and the Student’s *t*-test for independent (two-tailed) samples were used to compare categorial and continuous variables with ExPH, respectively. Pearson’s correlation analysis was used to compare the CV score with continuous factors, while the Student’s *t*-test for independent samples (two-tailed) was used to compare the CV score with categorical factors. All analyses were performed with the SPSS v.26 statistical software, with statistical significance set at 0.05.

## 3. Results

We enrolled 54 patients from January 2020 to July 2021 in the outpatient clinic dedicated to the diagnosis and treatment of pulmonary hypertension, University Cardiology Division, University Hospital of Pisa.

**Figure 1:**
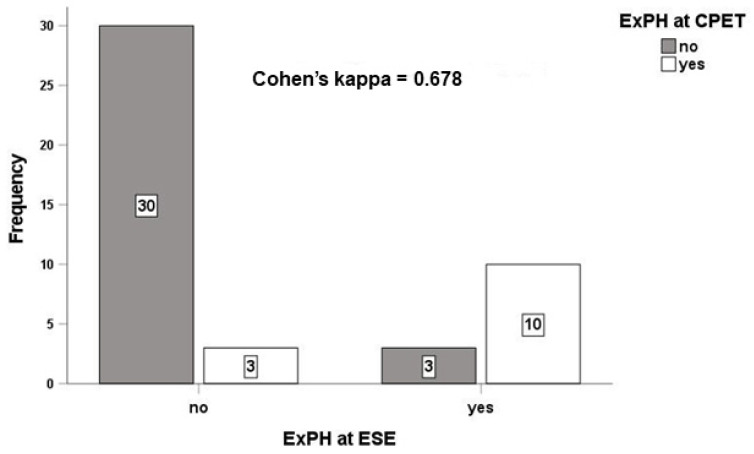
Concordance of exercise stress echocardiography and the cardiopulmonary exercise test in the diagnosis of isolated exercise pulmonary hypertension. Abbreviations: ESE, exercise stress echocardiogram; ExPH, exercise-induced pulmonary hypertension; CPET, cardiopulmonary exercise test.

All patients included in the study had no abnormalities on chest x-ray, lung function tests or electrocardiogram. We excluded eight patients with a high probability of PH on the resting echocardiogram. The remaining 46 were admitted to the study. Table A1 and Table A2 report the baseline characteristics, medical and drug histories of the entire study cohort with respect to the presence and absence of isolated ExPH at CPET and ESE, respectively. In our cohort, 72% (*n* = 33) of the enrolled population did not develop ExPH at ESE and 91% (*n* = 30) of these patients also did not develop ExPH at CPET, with a Cohen’s kappa = 0.678, indicating moderate agreement in the diagnosis of isolated ExPH between the two different methods (Figure 1). The mean age of the 46 participants included in the study was 53 ± 11. The age of those with ExPH diagnosed by CPET (Table A1) and ESE (Table A2) was 52 ± 14 years and 51 ± 13 years, respectively, while the age of those without ExPH at CPET and ESE was 54 ± 10 and 55 ± 10, respectively. We observed no statistically significant differences in patients with and without ExPH on CPET and ESE in terms of mean body surface index (BSA) age, sex, body mass index (BMI), heart rate, systolic and diastolic blood pressure, history of hypertension, comorbidities, CV risk factors and concomitant drug use, bio-humoral data and proinflammatory markers such as IL-6, erythrocyte sedimentation rate (ESR), C-reactive protein (CRP) and fibrinogen (Table A1 and Table A2). Patients diagnosed with ExPH, both on CPET (Table A1) and ESE (Table A2), had reduced functional capacity compared to patients without ExPH (*p* < 0.001), while they did not show significant differences in terms of morphology and function of the right or left ventricle (Table A3 and Table A4). Patients with ExPH had higher values of sPAP (*p* < 0.001), TRV (*p* = 0.048), right atrial area (*p* = 0.008) and pulmonary vascular resistance (velocity ratio Peak TR and VTI_RVOT_ > 0.20, *p* <0.003), compared to patients without ExPH (Table A4). We did not observe any correlation between the diagnosis of ExPH at CPET and the echocardiographic parameters associated with the pulmonary circulation and the right chambers, with the exception of sPAP (Table A3).

We evaluated the CV risk score in all 46 patients. The population’s overall CV risk score ranged from 8 (low CV risk) to 14 (high CV risk), with an average of 10.2 ± 2.4. The isolated ExPH diagnosed by both CPET and ESE was directly correlated to a higher CV score (*p* < 0.001) (Table 1). Patients with a higher CV score also had lower CD4+ T-cell counts (*p* = 0.001), a higher proportion of clinical progression to AIDS (*p* < 0.001) and a poor immunological response to anti-retroviral therapy (ART) (*p* = 0.035) (Table 2) and a higher right atrial area (*p* = 0.006) at rest TTE compared to patients with a lower CV risk score (Table 3). Furthermore, the proportion of patients with PVRI (ratio of Peak TR velocity to VTI_RVOT_) > 0.20 and higher CV score was increased compared to patients with a lower CV score (*p* = 0.003), indicating higher total pulmonary vascular resistance in patients with higher CV risk (Table 3). No associations with time to HIV diagnosis, time to beginning ART, ART discontinuation, virological response to ART, current use of protease inhibitors and proinflammatory markers such as IL-6, ESR, CRP and fibrinogen were found (Table 2).

## 4. Discussion

We investigated whether isolated ExPH is associated with a higher CV risk score in patients with HIV without a high probability of PH at resting echocardiogram. We have found that patients who develop ExPH have a higher CV risk score, as assessed by the ESC/ERS Guidelines [1]. We have found that isolated ExPH is associated with worse WHO-FC, shorter walk distance at the 6MWT and a lower VO_2_ peak, indicating worse functional capacity. The worst CV risk score was actually clustered into the isolated ExPH group, suggesting the usefulness of ExPH in assessing CV risk of patients with HIV. This is supported by the fact that patients with a worse CV risk score who also had isolated ExPH showed worse echo parameters related to PH and its consequences on the right cardiac chambers, as these patients had a higher total pulmonary vascular resistance and a higher right atrial area at rest TTE. Our results suggest that the work-out of ExPH using the combined ESE and CPET approach allows for the identification of those patients with HIV who develop ExPH due to reduced pulmonary vasodilatory reserve and who have a worse CV risk profile.

Unlike the previous study, where we only used the echocardiographic approach in diagnosing ExPH [12], here we used a combined CPET-ESE approach to non-invasively assess cardiovascular and pulmonary responses to exercise. Several studies have shown that in different clinical settings, CPET is more sensitive and specific than ESE in identifying pulmonary vascular abnormalities precipitated by exercise [22]. In our cohort we could not evaluate the differential performance of CPET and ESE in detecting ExPH as we did not perform right stress cardiac catheterization, which is the reference gold standard [1]. However, we have shown that there is good agreement in diagnosis between the two different methods, as demonstrated by the high Cohen’s kappa index. However, the benefit of performing a combined CPET-ESE is the identification of concomitant left heart disease as the cause of exertional dyspnea and ExPH. Increased left ventricle filling pressure is known to lead to pulmonary venous congestion and post-capillary pulmonary hypertension, regardless of LVEF [23,24]. In our cohort, TEE at rest and ESE ruled out the existence of left heart disease as a possible cause of ExPH, regardless of the method used for diagnosis.

We have recently shown that the onset of ExPH, as diagnosed by ESE, is associated with poor immunological control of HIV infection [11]. Here we have found that those patients who have worse immunological control of the disease also have a higher CV risk score. Finally, patients with a higher CV risk score also had greater clinical progression to AIDS and poorer immunological response to ART. This confirms previous studies demonstrating the role of a weakened or dysfunctional immune system in determining CV risk and prognosis of HIV-related PAH [2,25,26]. Thus, isolated ExPH can identify patients with HIV at higher CV risk as a consequence of poorer immune control of the disease. PAH often complicates HIV infection and this leads to the increased mortality of these patients. We are now demonstrating that the development of isolated ExPH in such patients without a high PH probability at rest can be considered an early marker of a worsening outcome.

HIV proteins can trigger an inflammatory response, leading to PAH [27]. Patients with HIV have elevated levels of interleukin 6 (IL-6), tumor necrosis factor α (TNFα) and nitric oxide synthase (NO) inhibitors [28,29]. The inflammation theory of PAH is biologically and clinically plausible, as PAH continues to manifest significantly in patients with HIV as an expression of the persistence of this inflammatory component, despite a good response to ART [30]. However, so far there are no clinical studies that correlate high levels of these proinflammatory mediators with the development of PAH in patients with HIV. An exploratory Phase 1 clinical study, the first of its kind to our knowledge, is still ongoing to test the effects of a combination of the HIV protease inhibitors saquinavir and ritonavir on proinflammatory mediators and pulmonary hemodynamics in patients with idiopathic PAH (ClinicalTrials.gov identifier: NCT02023450). Therefore, the blood levels of these mediators may currently be useful biomarkers in the stratification of patients with HIV with a higher atherosclerotic CV risk, but not those who have a propensity to develop HIV-related PAH. On the other hand, these biomarkers are not included in any of the validated risk scores in patients with PAH group 1, including patients with HIV. In our cohort, we did not observe any significant association between levels of IL-6 or other proinflammatory markers and ExPH or a worse CV risk score. However, only a few patients had such biomarkers evaluated. Further investigation with more patients is needed to establish the role of proinflammatory biomarkers in the development of PAH and a worse CV risk score.

### Study Limitations

This study has several limitations: (1) the small sample size, for which our report should be intended as a pilot study on the role of ExPH in risk stratification of patients with HIV; (2) patients with ExPH at ESE and CPET did not undergo cardiac catheterization, which is, however, not indicated, and therefore unethical in such patients with low and intermediate probability of PH [1]. Finally, adequate follow-up could allow us to verify if patients with ExPH develop PH at rest, and if this is associated with a worsening of CV risk over time. Our research group is carrying out a long-term follow-up, which will help to clarify the prognostic significance of ExPH in the HIV population.

**In conclusion**: Isolated ExPH associates with a higher CV risk score in patients with HIV. Assessment of ExPH by CPET or ESE can contribute to the risk stratification of patients with HIV.

## Figures and Tables

**Table 1:** Comparison between score and ExPH.

	CV Score	*p*-Value
**ExPH at CPET**		<0.001
*no*	8.8 (0.7)	
*yes*	12.9 (2.1)	
**ExPH at ESE**		<0.001
*no*	8.9 (1.1)	
*yes*	12.5 (2.4)	

CV score values are shown as mean (SD). Abbreviations: ExPH, isolated exercise pulmonary hypertension; CPET, cardiopulmonary exercise test; ESE, exercise stress echocardiogram.

**Table 2:** Comparison between score and virological and immunological factors.

	Statistics	*p*-Value
**Time to HIV diagnosis (y)**	−0.155	0.309
**CD4+ T-cell count at diagnosis (cell/mmc)**	−0.402	0.012
**CD4+ T-cell count at diagnosis (%)**	−0.380	0.020
**CD4+ T-cell count last determination (cell/mmc)**	−0.386	0.009
**CD4+ T-cell count last determination (%)**	−0.480	0.001
**Clinical progression to AIDS**		<0.001
*no*	8.8 (1.1)	
*yes*	12.9 (2)	
**Development of resistance to ART**		0.451
*no*	9.8 (2.1)	
*yes*	10.4 (2.7)	
**HIV-RNA levels at diagnosis (cp/mL)**	−0.116	0.502
**HIV-RNA levels last determination (cp/mL)**	0.010	0.949
**HIV-RNA levels last determination (cp/mL)**		0.949
*<20 cp/mL*	9.9 (2.3)	
*>20 cp/mL*	10 (2.3)	
**Time to beginning of ART**	−0.182	0.226
**Current use of protease inhibitors**		0.101
*no*	9.8 (2.2)	
*yes*	11.5 (2.7)	
**Combination of ART with booster (ritonavir or cobicistat)**		0.827
*no*	10.1 (2.4)	
*yes*	9.9 (2.4)	
**Virologic response to ART**		0.690
*<20 copies/mL*	9.7 (2.2)	
*20–50 copies/mL*	10.1 (2.3)	
*50–200 copies/mL*	11.5 (3.5)	
*>200 copies/mL*	10.7 (2.9)	
**Immunologic response to ART**		0.035 *
*optimal*	9.3 (1.8)	
*acceptable*	10.6 (2.6)	
*not acceptable*	12.3 (2.9)	
*ART discontinuation*		0.536
*no*	10.2 (2.4)	
*yes*	9.7 (2.3)	
**IL-6**	0.289	0.152
**PCR**	−0.247	0.224
**VES**	0.194	0.354
**FBG**	−0.053	0.789

Statistics: Pearson’s r or mean (SD). Abbreviations: HIV, Human immunodeficiency virus; ART, antiretroviral therapy; IL-6, interleukin-6; PCR, reactive C protein; VES, Erythrocyte sedimentation rate; FBG, fibrinogen. * only the comparison between “optimal” and “not acceptable” was statistically significant with a *p* = 0.040, by Dunnett correction using “not acceptable” as the reference category.

**Table 3:** Comparison between score and ultrasound parameters.

Parameter	Statistics	*p*-Value
**Concentric remodeling**		0.557
*no*	9.9 (2.2)	
*yes*	10.3 (2.6)	
**Normal geometry**		0.755
*no*	10.1 (2.4)	
*yes*	9.9 (2.3)	
**Concentric hypertrophy**		0.014
*no*	10.2 (2.4)	
*yes*	8.7 (0.6)	
**Eccentric hypertrophy**		0.383
*no*	10 (2.3)	
*yes*	11.5 (3.5)	
**LAD**	−0.055	0.718
**LAV**	−0.076	0.620
**iLAV**	−0.067	0.667
**LVEF**	−0.164	0.281
**FwSV**	−0.097	0.527
**iFwSV**	−0.112	0.465
**MR**	−0.102	0.503
**AO**	−0.146	0.339
**MS**	−0.069	0.651
**AS**	−0.069	0.651
**E wave**	0.116	0.448
**A wave**	0.153	0.315
**Septal e wave**	0.025	0.871
**E/A**	−0.107	0.485
**E/e**	0.178	0.242
**iRVESA**	0.133	0.385
**iRVEDA**	0.069	0.652
**iRVESV**	0.124	0.419
**iRVEDV**	−0.133	0.385
**RD1**	0.075	0.626
**RD2**	−0.215	0.157
**RD3**	−0.275	0.068
**RVOT prox.**	−0.081	0.596
**RVOT dist.**	0.071	0.644
**Eccentricity index**	−0.207	0.171
**RV/LV basal diameter ratio**	−0.192	0.205
**TAPSE**	0.088	0.564
**FAC**	0.179	0.240
**RV E/A**	−0.140	0.358
**RV E/e**	−0.026	0.869
**RV S vel**	0.151	0.322
**RV S VTI**	−0.245	0.104
**TR**	−0.127	0.407
**sPAP**	−0.018	0.906
**aPAP at exercise peak**	0.269	0.074
**mPAP**	0.036	0.814
**TRV**	0.115	0.454
**TRV at exercise peak**	0.032	0.834
**RV outflow AT**	0.207	0.173
**VCI diameter**	−0.048	0.753
**RA area**	0.401	0.006
**iRAV**	0.273	0.070
**VTI at rvot**	−0.583	0.000
**VRT/VTI at rvot**	0.197	0.196
**TAPSE/PAPs**	0.101	0.508
**PVRI**		0.003
*<0.20*	8.88 (1.23)	
*>0.20*	12.10 (2.55)	

Statistics: Pearson’s r or mean (SD). Abbreviations: LVEDD, left ventricle end-diastolic diameter; LVESD, left ventricle end-systolic diameter; LVEDV, left ventricle end-diastolic volume; LVESV, left ventricle end-systolic volume; iLVEDV, indexed LVEDV; iLVESV, indexed LVESV; LAD, left atrial diameter; LAV, left atrial volume; iLAV, indexed LAV; LVEF, left ventricle ejection fraction; FwSV, forward stroke volume; iFwSV, indexed FwSV; iRVESA, indexed right ventricle end-systolic area; iRVEDA, indexed right ventricle end-diastolic area; iRVESV, indexed right ventricle end-systolic volume; iRVEDV, indexed right ventricle end-diastolic volume; RD, right diameter; RVOT, right ventricle outflow; TAPSE, tricuspid annular plane excursion; FAC, fractional area change; VTI, velocity time integral; sPAP, systolic pulmonary arterial pressure; mPAP, mean PAP; TR, tricuspid regurgitation; IVC, inferior vena cava; iRAV, indexed right atrial volume; TRV, tricuspid regurgitation velocity; PVRI, Pulmonary Vascular Resistance Index.

## Data Availability

The data presented in this study are available on request from the corresponding author.

## Supplementary Materials

The following supporting information can be downloaded at: https://www.mdpi.com/article/10.3390/jcm11092447/s1, online text: methods.

## Appendix A

**Table A1:** Demographics, medical history and medication use according to presence and absence of ExPH at CPET.

Clinical Parameter	ExPH at CPET	Mean	SD	*p*-Value
Age	*no*	54.21	9.76	0.615
	*yes*	52.38	13.86	
BSA	*no*	1.91	0.20	0.475
	*yes*	1.86	0.24	
SAP	*no*	126.36	12.20	0.652
	*yes*	124.62	10.50	
DAP	*no*	74.24	7.08	0.745
	*yes*	75.00	7.07	
		**ExPH at CPET**		
		**no**	**yes**	
Sex	F	11	5	0.742
	M	22	8	
Smoke	no	16	3	0.264
	yes	16	9	
Functional class FC-WHO	I	32	3	<0.001
	II	1	0	
	III	0	10	
Chronic liver disease	*no*	16	8	0.425
	*yes*	17	5	
Kidney disease	*no*	29	12	0.664
	*yes*	4	1	
Thyroid disease	*no*	29	11	0.767
	*yes*	4	2	
Arterial hypertension	*no*	29	13	0.189
	*yes*	4	0	
Dyslipidemia	*no*	10	6	0.309
	*yes*	23	7	
Diabetes mellitus	*no*	29	11	0.767
	*yes*	4	2	
Familiarity for CAD	*no*	26	10	0.891
	*yes*	7	3	
Calcium channel blockers	*no*	29	12	0.206
	*yes*	4	0	
Hypoglycemic drugs	*no*	10	6	0.309
	*yes*	23	7	
Beta blockers	*no*	32	13	0.526
	*yes*	1	0	
ACE inhibitors or sartanics	*no*	32	13	0.526
	*yes*	1	0	
Thyroid hormones	*no*	29	11	0.767
	*yes*	4	2	
Lipid lowering drugs	*no*	10	6	0.309
	*yes*	23	7	

Abbreviations: ExPH, isolated exercise pulmonary hypertension; CPET, cardiopulmonary exercise test; BSA, body surface area; SAP, systolic arterial pressure; DAP, diastolic arterial pressure; CAD, coronary artery disease; F, female; M, male; ACE, angiotensin converting enzyme.

**Table A2:** Demographics, medical history and medication use according to presence and absence of ExPH at ESE.

Factor	ExPH at ESE	Mean	SD	*p*-Value
Age	*no*	54.58	9.94	0.391
	*yes*	51.46	13.33	
BSA	no	1.90	0.20	0.932
	*yes*	1.89	0.25	
Heart rate	*no*	69.03	6.30	0.839
	yes	68.62	5.98	
	yes	126.92	9.47	
DAP	no	73.79	7.18	0.308
	yes	76.15	6.50	
		**ExPH at ESE**		
**Factor**		**no**	**yes**	***p*-value**
Sex	F	12	4	0.720
	M	21	9	
Smoke	no	15	4	0.570
	ex	1	1	
	yes	17	8	
Functional class FC-WHO	I	31	4	<0.001
	II	1	0	
	III	1	9	
Chronic liver disease	*no*	15	9	0.146
	*yes*	18	4	
Kidney disease	*no*	29	12	0.664
	*yes*	4	1	
Thyroid disease	*no*	28	12	0.499
	*yes*	5	1	
Arterial hypertension	*no*	30	12	0.880
	*yes*	3	1	
Dyslipidemia	*no*	9	7	0.088
	*yes*	24	6	
Diabetes mellitus	*no*	28	12	0.499
	*yes*	5	1	
Familiarity for CAD	*no*	27	9	0.351
	*yes*	6	4	
Calcium channel blockers	*no*	30	11	0.937
	*yes*	3	1	
Hypoglycemic drugs	*no*	9	7	0.088
	*yes*	24	6	
Beta blockers	*no*	33	12	0.107
	*yes*	0	1	
ACE inhibitors or sartanics	*no*	32	13	0.526
	*yes*	1	0	
Thyroid hormones	*no*	28	12	0.499
	*yes*	5	1	
Lipid lowering drugs	*no*	9	7	0.088
	*yes*	24	6	

Abbreviations: ExPH, isolated exercise pulmonary hypertension; ESE, exercise stress echocardiography; BSA, body surface area; F, female; M, male; CAD, coronary artery disease; ACE, angiotensin converting enzyme.

**Table A3:** ExPH at CPET vs. continuous or categorical ultrasound parameters.

Continuous Ultrasound Parameters	ExPH at CPET	Mean	SD	*p*-Value
LVEDD	*no*	43.656	5.522	0.959
	*yes*	43.558	5.978	
LVESD	*no*	26.656	6.439	0.442
	*yes*	24.917	7.090	
LVEDV	*no*	103.875	31.270	0.299
	*yes*	93.000	28.451	
iLVEDV	*no*	36.676	10.597	0.163
	*yes*	31.559	10.808	
LVESV	*no*	38.219	14.041	0.314
	*yes*	33.167	16.219	
iLVESV	*no*	13.416	4.944	0.384
	*yes*	11.923	5.184	
LV	*no*	152.884	48.281	0.443
	*yes*	140.817	38.913	
iLVmass	*no*	79.132	29.520	0.255
	*yes*	68.110	24.111	
LAD	*no*	48.359	8.571	0.919
	*yes*	48.083	5.744	
LAV	*no*	38.778	20.368	0.294
	*yes*	32.317	7.786	
iLAV	*no*	13.683	7.657	0.468
	*yes*	12.000	3.149	
LVEF	*no*	65.147	6.406	0.584
	*yes*	66.500	9.200	
FwSV	*no*	72.366	22.197	0.268
	*yes*	63.750	24.000	
iFwSV	*no*	25.367	7.810	0.195
	*yes*	21.745	8.944	
MR	*no*	0.688	0.780	0.027
	*yes*	0.250	0.452	
AO	*no*	0.250	0.672	1.000
	*yes*	0.250	0.622	
E wave	*no*	69.769	17.989	0.993
	*yes*	69.817	14.658	
A wave	*no*	71.397	30.384	0.783
	*yes*	74.167	26.889	
Septal e wave	*no*	9.959	3.003	0.837
	*yes*	9.767	1.837	
E/A	*no*	1.067	0.394	0.909
	*yes*	1.085	0.629	
E/e	*no*	6.458	3.408	0.299
	*yes*	7.575	2.193	
iRVESA	*no*	4.055	1.879	0.396
	*yes*	4.578	1.542	
iRVEDA	*no*	6.955	2.143	0.991
	*yes*	6.947	2.343	
iRVESV	*no*	7.161	4.907	0.908
	*yes*	7.338	2.902	
iRVEDV	*no*	16.048	6.550	0.220
	*yes*	13.507	4.273	
RD1	*no*	36.128	7.270	0.822
	*yes*	36.667	6.372	
RD2	*no*	33.472	5.483	0.116
	*yes*	30.333	6.513	
RD3	*no*	20.503	6.485	0.041
	*yes*	16.083	5.351	
RVOT prox.	*no*	31.716	6.057	0.627
	*yes*	30.792	3.905	
RVOT dist.	*no*	32.353	7.471	0.166
	*yes*	29.042	5.163	
eccentricity index	*no*	0.941	0.110	0.475
	*yes*	0.914	0.103	
RV/LV basal diameter ratio	*no*	0.864	0.162	0.317
	*yes*	0.808	0.162	
TAPSE	*no*	21.913	7.104	0.707
	*yes*	22.842	7.665	
FAC	*no*	45.053	9.607	0.210
	*yes*	49.083	8.554	
RV E/A	*no*	4.338	13.318	0.389
	*yes*	0.963	0.408	
RV E/e	*no*	3.673	1.829	0.683
	*yes*	3.938	1.891	
RV S vel	*no*	10.993	4.485	0.630
	*yes*	11.767	5.301	
RV S VTI	*no*	3.608	4.050	0.024
	*yes*	1.810	0.926	
TR	*no*	1.219	0.491	0.862
	*yes*	1.250	0.622	
sPAP	*no*	22.375	5.752	0.749
	*yes*	21.750	5.675	
sPAP at exercise peak	*no*	32.847	10.602	0.028
	*yes*	40.767	9.452	
mPAP	*no*	16.333	3.623	0.969
	*yes*	16.385	4.636	
TRV	*no*	2.009	0.373	0.622
	*yes*	2.075	0.449	
TRV at exercise peak	*no*	2.544	0.555	0.557
	*yes*	2.658	0.616	
RV outflow AT	*no*	102.714	42.717	0.014
	*yes*	138.167	34.821	
VCI diameter	*no*	15.016	3.777	0.571
	*yes*	14.158	5.896	
RA area	*no*	14.709	3.227	0.295
	*yes*	15.875	3.294	
iRAV	*no*	5.356	2.191	0.410
	*yes*	5.924	1.420	
VTI at rvot	*no*	15.843	3.811	<0.001
	*yes*	11.167	2.916	
VRT/VTI at rvot	*no*	0.156	0.142	0.414
	*yes*	0.191	0.051	
TAPSE/PAPs	*no*	1.033	0.347	0.609
	*yes*	1.100	0.471	
**Categorical ultrasound parameters**	**ExPH at ESE**	***no***	***yes***	***p*-value**
Concentric remodeling	*no*	19	5	0.293
	*yes*	13	7	
Normal geometry	*no*	18	7	0.901
	*yes*	14	5	
Concentric hypertrophy	*no*	29	12	0.272
	*yes*	3	0	
Eccentric hypertrophy	*no*	31	12	0.536
	*yes*	1	0	

Abbreviations: ExPH, isolated exercise pulmonary hypertension; CPET, cardiopulmonary exercise test; LVEDD, left ventricle end-diastolic diameter; LVESD, left ventricle end-systolic diameter; LVEDV, left ventricle end-diastolic volume; LVESV, left ventricle end-systolic volume; iLVEDV, indexed LVEDV; iLVESV, indexed LVESV; LAD, left atrial diameter; LAV, left atrial volume; iLAV, indexed LAV; LVEF, left ventricle ejection fraction; FwSV, forward stroke volume; iFwSV, indexed FwSV; iRVESA, indexed right ventricle end-systolic area; iRVEDA, indexed right ventricle end-diastolic area; iRVESV, indexed right ventricle end-systolic volume; iRVEDV, indexed right ventricle end-diastolic volume; RD, right diameter; RVOT, right ventricle outflow; TAPSE, tricuspid annular plane excursion; FAC, fractional area change; VTI, velocity time integral; sPAP, systolic pulmonary arterial pressure; mPAP, mean PAP; TR, tricuspid regurgitation; IVC, inferior vena cava, iRAV, indexed right atrial volume; TRV, tricuspid regurgitation velocity; ESE, exercise stress echocardiogram.

**Table A4:** ExPH at ESE vs. continuous or categorical ultrasound parameters.

Continuous Ultrasound Parameters	ExPH at ESE	Mean	SD	*p*-Value
LVEDD	*no*	43.459	5.322	0.745
	*yes*	44.083	6.445	
LVESD	*no*	26.625	6.460	0.472
	*yes*	25.000	7.058	
LVEDV	*no*	100.938	31.460	0.992
	*yes*	100.833	29.489	
iLVEDV	*no*	35.857	10.955	0.568
	*yes*	33.743	10.596	
LVESV	*no*	36.406	14.555	0.752
	*yes*	38.000	15.486	
iLVESV	*no*	12.992	5.205	0.971
	*yes*	13.054	4.604	
LV	*no*	149.453	44.358	0.974
	*yes*	149.967	51.515	
iLV mass	*no*	77.544	28.061	0.593
	*yes*	72.343	29.882	
LAD	*no*	48.531	8.481	0.737
	*yes*	47.625	6.057	
LAV	*no*	38.844	19.955	0.276
	*yes*	32.142	10.252	
iLAV	*no*	13.895	7.507	0.290
	*yes*	11.452	3.692	
LVEF	*no*	66.459	7.437	0.157
	*yes*	63.000	6.030	
FwSV	*no*	73.022	24.849	0.155
	*yes*	62.000	13.671	
iFwSV	*no*	25.663	8.952	0.090
	*yes*	20.957	4.347	
MR	*no*	0.656	0.787	0.193
	*yes*	0.333	0.492	
AO	*no*	0.313	0.738	0.304
	*yes*	0.083	0.289	
E wave	*no*	68.084	18.606	0.284
	*yes*	74.308	10.979	
A wave	*no*	71.400	30.367	0.784
	*yes*	74.158	26.943	
Septal e wave	*no*	10.128	2.958	0.384
	*yes*	9.317	1.908	
E/A	*no*	1.037	0.397	0.415
	*yes*	1.166	0.613	
E/e	*no*	6.251	3.400	0.077
	*yes*	8.129	1.778	
iRVESA	*no*	4.083	1.862	0.492
	*yes*	4.505	1.622	
iRVEDA	*no*	7.029	2.113	0.708
	*yes*	6.749	2.405	
iRVESV	*no*	7.183	4.886	0.950
	*yes*	7.278	3.001	
iRVEDV	*no*	16.217	6.461	0.126
	*yes*	13.058	4.326	
RD1	*no*	35.987	6.920	0.660
	*yes*	37.042	7.344	
RD2	*no*	33.188	5.538	0.297
	*yes*	31.092	6.712	
RD3	*no*	20.031	6.699	0.222
	*yes*	17.342	5.520	
RVOT prox.	*no*	31.119	5.782	0.505
	*yes*	32.383	4.882	
RVOT dist.	*no*	31.656	7.305	0.754
	*yes*	30.900	6.468	
Eccentricity index	*no*	0.943	0.112	0.335
	*yes*	0.908	0.096	
RV/LV basal diameter ratio	*no*	0.852	0.157	0.815
	*yes*	0.839	0.179	
TAPSE	*no*	21.707	6.949	0.495
	*yes*	23.392	7.958	
FAC	*no*	45.397	9.376	0.391
	*yes*	48.167	9.609	
RV E/A	*no*	4.333	13.319	0.391
	*yes*	0.976	0.431	
RV E/e	*no*	3.729	1.882	0.946
	*yes*	3.774	1.739	
RV S vel	*no*	10.950	4.497	0.561
	*yes*	11.883	5.251	
RV S VTI	*no*	3.174	3.328	0.867
	*yes*	2.968	4.289	
TR	*no*	1.313	0.471	0.077
	*yes*	1.000	0.603	
sPAP	*no*	22.344	6.003	0.794
	*yes*	21.833	4.896	
sPAP at exercise peak	*no*	30.909	8.846	<0.001
	*yes*	45.933	7.504	
mPAP	*no*	16.351	3.802	0.991
	*yes*	16.337	4.212	
TRV	*no*	2.009	0.389	0.622
	*yes*	2.075	0.409	
TRV at exercise peak	*no*	2.472	0.502	0.048
	*yes*	2.850	0.659	
RV outflow AT	*no*	105.027	43.990	0.065
	*yes*	132.000	36.352	
VCI diameter	*no*	14.722	4.534	0.885
	*yes*	14.942	4.191	
RA area	*no*	14.253	3.127	0.008
	*yes*	17.092	2.710	
iRAV	*no*	5.266	2.186	0.189
	*yes*	6.166	1.300	
VTI at rvot	*no*	16.065	3.552	<0.001
	*yes*	10.575	2.703	
VRT/VTI at rvot	*no*	0.153	0.143	0.289
	*yes*	0.198	0.041	
TAPSE/PAPs	*no*	1.024	0.321	0.448
	*yes*	1.123	0.516	
**Categorical ultrasound parameter**	**ExPH at ESE**	**no**	**yes**	***p*-value**
Concentric remodeling	*no*	19	5	0.293
	*yes*	13	7	
Normal geometry	*no*	18	7	0.901
	*yes*	14	5	
Concentric hypertrophy	*no*	29	12	0.272
	*yes*	3	0	
Eccentric hypertrophy	*no*	31	12	0.536
	*yes*	1	0	

Abbreviations: ExPH, isolated exercise pulmonary hypertension; ESE, exercise stress echocardiogram; LVEDD, left ventricle end-diastolic diameter; LVESD, left ventricle end-systolic diameter; LVEDV, left ventricle end-diastolic volume; LVESV, left ventricle end-systolic volume; iLVEDV, indexed LVEDV; iLVESV, indexed LVESV; LAD, left atrial diameter; LAV, left atrial volume; iLAV, indexed LAV; LVEF, left ventricle ejection fraction; FwSV, forward stroke volume; iFwSV, indexed FwSV; iRVESA, indexed right ventricle end-systolic area; iRVEDA, indexed right ventricle end-diastolic area; iRVESV, indexed right ventricle end-systolic volume; iRVEDV, indexed right ventricle end-diastolic volume; RD, right diameter; RVOT, right ventricle outflow; TAPSE, tricuspid annular plane excursion; FAC, fractional area change; VTI, velocity time integral; sPAP, systolic pulmonary arterial pressure; mPAP, mean PAP; TR, tricuspid regurgitation; IVC, inferior vena cava, iRAV, indexed right atrial volume; TRV, tricuspid regurgitation velocity.
