# Supplementary material for: Exercise-Induced Pulmonary Hypertension Is Associated with High Cardiovascular Risk in Patients with HIV
Source: J Clin Med. 2022 Apr 27;11(9):2447. doi: 10.3390/jcm11092447 (PMC9100247; doi:10.3390/jcm11092447)
Supplement: Supplementary file 1 [file jcm-11-02447-s001.zip › jcm-1675517-supplementary.pdf]

Exercise-induced pulmonary hypertension is associated with high cardiovascular risk in HIV patients

Short title: Exercise pulmonary hypertension in HIV

Rosalinda Madonna[1], Silvia Fabiani[2], Riccardo Morganti[3], Arianna Forniti[2], Filippo Biondi[1], Lorenzo Ridolfi[1], Riccardo Iapoce[2], Francesco Menichetti[2] and Raffaele De Caterina[1]

[1] Cardiology Division, Azienda Ospedaliera Universitaria Pisana and University of Pisa, Pisa, Italy

[2] Infectious Disease Unit, Department of Clinical and Experimental Medicine, Azienda Ospedaliera Universitaria Pisana, University of Pisa, Pisa, Italy

[3] Section of Statistics, University Hospital of Pisa, Pisa, Italy

Online supplement

Inclusion criteria and Data Collection

This was a prospective, observational, cohort study of 54 HIV patients recruited from the Infectious Disease Clinic at Pisa University Hospital. The study complies with the Helsinki Declaration, and informed consent was obtained from all patients before any diagnostic test, always performed for clinical purposes. The local investigators had full access to patient data and medical records.

All patients enrolled underwent transthoracic echocardiography (TTE) followed by a transthoracic exercise stress echocardiogram (ESE) and an exercise cardiopulmonary test (CPET). According to the TTE evaluation of PH probability [1,2], patients included had either a "low" PH probability at rest [defined as a tricuspidal regurgitation velocity (TRV) ≤2.8 m/s or not measurable, without additional PH signs, *n* = 43] or an "intermediate" PH probability at rest (TRV ≤2.8 m/s or not measurable, with additional PH signs; or TRV 2.9–3.4 m/s without additional PH signs, *n* = 3). We excluded patients with a "high" PH probability (TRV 2.9–3.4 m/s with additional PH signs, or TRV > 3.4 m/s, *n* = 8). Additional exclusion criteria were the presence of moderate-to-severe anemia (hemoglobin <10 g/dL); significant left heart disease at the resting TTE; history of venous thromboembolism; severe kidney disease; or chronic obstructive pulmonary disease – all possible additional causes of PH; and moderate to severe tricuspid insufficiency.

Isolated ExPH was defined as the absence of PH at rest and a raise of sPAP >20 mmHg during a low-intensity exercise (50 watts (W) over 2 minutes) not exceeding a cardiac output (heart rate x stroke volume) of 10 L/min [3,4,5]. Isolated ExPH at CPET was defined as a reduced oxygen uptake (peak VO_2); a minute ventilation relative to carbon dioxide production ratio (VE/VCO_2) >30 at the anaerobic threshold (AT); a reduced O_2 pulse <10 mL/beat at peak exercise; and a reduced amount of oxygen required at each workload ($\Delta VO_2/\Delta W$) <9 mL/min/W [6,7]. Patients were thus classified as either with or without ExPH at ESE or CPET. The two groups of patients were compared. We then evaluated the association of a higher CV score with the following prognostic determinants of cardiovascular (CV) risk, according to the 2015 European Society of Cardiology (ESC)/European Respiratory Society (ERS) guidelines [8]: 1. clinical signs of heart failure (absent/present); 2. syncope (absent/occasional/repeated); 3. worsening of World Health Organization Functional Class (WHO-FC) (I-II/III/IV); 4. deterioration of functional performance at CPET (peak VO_2 >15, peak VO_2 11-15, peak VO_2 <11; VE/VCO_2 slope <36, VE/VCO_2 slope 36–44.9, VE/VCO_2 slope ≥45); 5. deterioration of functional performance at the 6-Minute Walk test [(6MWT) >440 m, 165-440 m, <165 m); 6. echocardiographic parameters, including (a) the right atrial (RA) area (estimated

as <18 cm^2; 18-26 cm^2; or >26 cm^2); and (b) the extent of pericardial effusion (defined as absent, minimal, or moderate-to-severe). We assigned a severity score of 1-3 to each prognostic determinant and derived an overall CV risk score [9]. We also evaluated the association of a higher CV score with: (1) the time to HIV diagnosis; (2) the CD4+ T cell count, as an index indicator of immune function in HIV patients; (3) the clinical progression to acquired immunodeficiency syndrome (AIDS); (4) the development of resistance to antiretroviral therapy (ART); (5) HIV RNA levels (copies/mL); (6) the time to the start of ART; (7) the current use of protease inhibitors; (9) the combination of ART with booster drugs (ritonavir or cobicistat); (8) the immuno-virologic response to ART; (9) ART discontinuation; (10) pro-inflammatory markers such as interleukin-6 (IL-6), C-reactive protein (CRP), erythrocyte sedimentation rate (ESR), fibrinogen; (11) echocardiographic parameters [2].

Mono and 2D Transthoracic Echocardiography was performed using a Philips iE33 echocardiograph (Philips iE33 xMATRIX echocardiography system, Andover, MA) [13]. The images were recorded in at least three cardiac cycles. Right atrial pressure (RAP) was assessed by evaluating inferior vena cava (IVC) diameter and collassability during inspirium [13]. Sistolic pulmonary arterial pressure (PAPs) was calculated by adding RAP to the maximum systolic pressure gradient from tricuspid regurgitation velocity (TRV). Left atrial volume index (LAVi) was calculated by Simpson's algorithm in apical four-chamber and two-chamber view [13]. The following parameters were recorded in apical four-chamber and two-chamber views: left ventricular end-diastolic volume (LVEDV), left ventricular end-systolic volume (LVESV), left ventricular ejection fraction (LVEF), E and A wave velocity on Doppler of transmitral flow. LVEDV, LVESV and LVEF were calculated according to the Simpson biplane method. We assessed right ventricular function by measuring the tricuspid annular plane systolic excursion (TAPSE) and the fractional area change (FAC), according to the guidelines of the American Society of Echocardiography [10]. Since TAPSE and FAC can be load-dependent, we excluded from the study patients with moderate or severe tricuspid insufficiency. Mitral, aortic and tricuspidal regurgitation were assessed and, if they were more than mild, they were measured using the methods of the contracta vein and proximal isovelocity hemispheric surface area (PISA). Valvular diseases were classified from trivial to mild to moderate to severe according to current recommendations [10-12].

European Society of Cardiology echocardiographic probability grading for pulmonary hypertension

We assessed the peak TRV to determine the echocardiographic probability of PH according to the recently updated ESC/ERS PH guidelines [1,8] We sought echocardiographic PH signs, classified as present if at least 2 of the 3 categories (A–C) were documented: (A) right ventricle/left ventricle basal diameter ratio >1.0; flattening of the interventricular septum (left ventricular eccentricity index >1.1 in systole and/or diastole); (B) right ventricular acceleration time <105 ms and/or the presence of a mid-systolic notching; early diastolic pulmonary regurgitation velocity >2.2 m/s; pulmonary artery diameter >25 mm; (C) inferior vena cava diameter >21 mm with decreased inspiratory collapse (<50% with a sniff or <20% with quiet inspiration); right atrial area (end-systole) >18 cm^2.

Stress echocardiography

All patients underwent a semi-supine ESE performed with a 2.5-MHz duplex transducer and conventional ultrasound system (Philips, Milan, Italy) on a semi-recumbent cycle ergometer (Ergoline, model 900 EL, Germany), according to the protocol recommended by the European Association of Echocardiography (EAE) guidelines [13]. Graded cycling was performed starting at an initial workload of 25 W lasting for 2 min. The electrocardiogram (ECG) and blood pressure were continuously monitored. Criteria for interrupting the test were the occurrence of severe chest pain, a diagnostic ST-segment shift, fatigue, an excessive blood pressure increase (systolic blood pressure ≥240 mmHg, diastolic blood pressure ≥120 mmHg), limiting dyspnea, or maximal predicted heart rate. We also evaluated the maximum rate-pressure product (heart rate x systolic blood pressure) and exercise time (in min). We performed echocardiographic imaging from the parasternal long-axis, the short-axis, the four-chamber and the three-chamber views.

Cardiopulmonary Exercise Test

We performed a symptom-limited CPET on an electronically-braked cycle ergometer, performed with Vmax 6200 Spectra Series (SensorMedics, Hoechberg, Germany), using a ramp-pattern increase in work rate. After calibration of the volumes and gas exchange analyzers, subjects breathed through a Rudolph mask connected with a two-way respiratory valve. We stopped the exercise test when one or more of the following criteria were present: achievement of the predicted heart rate; fatigue; dyspnea; excessive arterial

blood pressure increase (>230/130 mmHg); ≥2 mm ST-segment depression in at least two adjacent leads; and/or angina. We considered achievement of the ischemic threshold as the onset of a 1 mm ST-segment depression in at least two adjacent leads [14]. We measured the AT with the V-slope method [6,7]. Peak oxygen uptake (VO_2 peak) was the average oxygen uptake during the last 15 s of exercise, $\%VO_2$ max was the percent of VO_2 peak predicted by anthropometric data, and % AT was the percentage of predicted AT. We defined O_2 pulse as the oxygen uptake divided by the hear rate (HR), and % O_2 pulse as the percent of predicted O_2 pulse [6,7], VE/VCO_2 and VE/VO_2 were the slope of ventilation versus CO_2 output and O_2 uptake, respectively. The $\Delta VO_2/\Delta W$ slope was automatically calculated.

References

1. Madonna R, Bonitatibus G, Vitulli P, Pierdomenico SD, Galie N, De Caterina R. Association of the European Society of Cardiology echocardiographic probability grading for pulmonary hypertension with short and mid-term clinical outcomes after heart valve surgery. Vascul Pharmacol 2020;125-126:106648.
2. Madonna R, Fabiani S, Morganti R, et al. Exercise-induced pulmonary hypertension in HIV patients: Association with poor clinical and immunological status. Vascul Pharmacol 2021;139:106888.
3. Borlaug BA, Nishimura RA, Sorajja P, Lam CS, Redfield MM. Exercise hemodynamics enhance diagnosis of early heart failure with preserved ejection fraction. Circ Heart Fail 2010;3:588-595.
4. Nagel C, Henn P, Ehlken N, et al. Stress Doppler echocardiography for early detection of systemic sclerosis-associated pulmonary arterial hypertension. Arthritis Res Ther 2015;17:165.
5. Argiento P, Chesler N, Mule M, et al. Exercise stress echocardiography for the study of the pulmonary circulation. Eur Respir J 2010;35:1273-1278.
6. Belardinelll R. Il Test da Sforzo Cardiopolmonare. Monza, Italy: Midia; 2006.
7. Wassermann K. Cardiopulmonary Exercise Testing and Cardiovascular Health. Hoboken, NJ, USA: Wiley-Blackwell; 2002.
8. Galie N, Humbert M, Vachiery JL, et al. 2015 ESC/ERS Guidelines for the diagnosis and treatment of pulmonary hypertension: The Joint Task Force for the Diagnosis and Treatment of Pulmonary Hypertension of the European Society of Cardiology (ESC) and the European Respiratory Society (ERS): Endorsed by: Association for European Paediatric and

Congenital Cardiology (AEPC), International Society for Heart and Lung Transplantation (ISHLT). Eur Heart J 2016;37:67-119.
9. Madonna R, Morganti R, Radico F, et al. Isolated Exercise-Induced Pulmonary Hypertension Associates with Higher Cardiovascular Risk in Scleroderma Patients. J Clin Med 2020;9.
10. Rudski LG, Lai WW, Afilalo J, et al. Guidelines for the echocardiographic assessment of the right heart in adults: a report from the American Society of Echocardiography endorsed by the European Association of Echocardiography, a registered branch of the European Society of Cardiology, and the Canadian Society of Echocardiography. J Am Soc Echocardiogr 2010;23:685-713; quiz 786-688.
11. Baumgartner H, Hung J, Bermejo J, et al. Echocardiographic assessment of valve stenosis: EAE/ASE recommendations for clinical practice. J Am Soc Echocardiogr 2009;22:1-23; quiz 101-102.
12. Cagnina A, Michaux S, Ancion A, Cardos B, Damas F, Lancellotti P. [European guidelines for the diagnosis and management of acute pulmonary embolism]. Rev Med Liege 2021;76:208-215.
13. Sicari R, Nihoyannopoulos P, Evangelista A, et al. Stress echocardiography expert consensus statement: European Association of Echocardiography (EAE) (a registered branch of the ESC). Eur J Echocardiogr 2008;9:415-437.
14. Badesch DB, Champion HC, Gomez Sanchez MA, et al. Diagnosis and assessment of pulmonary arterial hypertension. J Am Coll Cardiol 2009;54:S55-S66.
